# Correlation between negative life events and suicide attempts among Yi adolescents with HIV/AIDS in Liangshan Prefecture

**DOI:** 10.1186/s12889-023-16377-1

**Published:** 2023-08-22

**Authors:** Xiaozhen Song, Shoukang Zou, Yangling Li, Liyu Chen, Ping Feng, Peiwei Xu, Hang Zhang, Fang Deng, Hanmei Xu, Yuanmei Tao, Meijiang Jin, Maojia Ran, Hong Zhang, Fanghua Ma, Ying Wang, Li Yin

**Affiliations:** 1 Mental Health Center, West China Hospital, Sichuan University, Chengdu, China; 2 The Fourth People’s Hospital of Chengdu, Chengdu, China; 3 Center for Infectious Diseases, West China Hospital, Sichuan University, Chengdu, China; 4 Antiviral Treatment Center, Zhao jue County People’s Hospital, Shenzhen, China

**Keywords:** Adolescents with HIV/AIDS, Liangshan Prefecture, Yi nationality, Negative life events, Suicide attempts

## Abstract

**Objective:**

To investigate the incidence of suicide attempts among adolescents with HIV/AIDS in Liangshan Prefecture, Sichuan Province, as well as the correlation between negative life events, sleep, exercise, drug therapy and suicide attempts.

**Methods:**

A total of 180 Yi adolescents aged 11–19 years with HIV/AIDS in a county of Liangshan Prefecture, Sichuan Province, China, were investigated by census. The main outcome indicators included the incidence of suicide attempts and whether negative life events, sleep, exercise, drug therapy and other factors were related to suicide attempts.

**Results:**

We found that the incidence rate of suicide attempts among Yi adolescents with HIV/AIDS in Liangshan Prefecture was 13.9%. Negative life events were a risk factor for suicide attempts (OR = 1.047, p < 0.001, 95% CI 1.027–1.067). In the factors of negative life events, adaptation was a risk factor for suicide attempts (OR = 1.203, p = 0.026, 95% CI 1.022–1.416), and academic pressure showed a tendency to be a risk factor for suicide attempts (OR = 1.149, p = 0.077, 95% CI 0.985–1.339). However, the punishment factor, interpersonal stress factor and loss factor had no significant correlation with suicide attempts. There was no significant correlation between sleep, exercise, drug therapy and suicide attempts.

**Conclusion:**

The proportion of suicide attempts among Yi adolescents with HIV/AIDS in Liangshan Prefecture is high and should be considered. Negative life events are independent risk factors for suicide attempts, and it is necessary to strengthen the screening and early intervention for suicide attempts in HIV/AIDS adolescents with definite negative life events.

## Introduction

Suicide is a serious threat to adolescents and needs to be paid sufficient attention by all walks of life. Previous studies have found that the incidence of suicide attempts among adolescents is approximately 5.9–17%, and the risk factors for suicide attempts include negative life events, no longer going to school, smoking, bullying, violence, etc. [1–5]., and high family cohesion and peer support are considered protective factors for suicide attempts [6]. In terms of gender differences, many studies have found that the incidence of suicide attempts among girls is significantly higher than that among boys [7–10]. In addition, according to data from the World Health Organization (WHO), suicide is the fourth leading cause of death among teenagers aged 15–29 globally [11], so investigation and research on the incidence of suicide attempts and its influencing factors can provide more reliable and scientific evidence for suicide prevention.

Different sleep and exercise conditions may have different effects on adolescents’ mental health and suicide attempts. Sleep problems such as lack of sleep and poor sleep quality are generally associated with a higher risk of suicide attempts in adolescents [12–14]. However, the correlation between exercise and adolescent suicide attempts has somewhat different results. The study conducted by Mireia Felez-Nobrega showed that adolescents who met the amount of recommended exercise by the WHO among boys were associated with a lower risk of suicide attempts, while those among girls were associated with a higher risk of suicide attempts [15]. However, Chung Gun Lee’s study in South Korea did not find that physical exercise could reduce the risk of suicide attempts and found that frequent vigorous physical exercise increased the risk of suicide attempts [16].

Many studies have found that the incidence of suicide attempts among ethnic minority adolescents is significantly higher than that among local adolescents [17–19], and emotional problems, sexual abuse and social anxiety disorder are risk factors for suicide attempts among ethnic minority adolescents [18, 20]. Myriam Forster performed a longitudinal study on Latino adolescents in California and found that the group’s emotional identity with race was a protective factor against suicide attempts [21]. Bridget A. Nestor found that non-Hispanic black and Native American adolescents in the United States were significantly less likely than white adolescents to use health services for suicide [22].

HIV/AIDS is a serious threat to human health; at the same time, suicide should be given special attention among HIV/AIDS patients, and it is also obviously related to depression and other mental diseases. A cohort study conducted in New York by Philip Kreniske found that adolescents with HIV had a significantly increased risk of attempted suicide compared with adolescents exposed to perinatal HIV but not infected with HIV [23]. A meta-analysis of 14 studies from 7 countries conducted by Light Tsegay showed that the lifetime prevalence of suicide attempts among adolescents with HIV was 13.05% [24]. Risk factors include high depression score, behavior disorder, low socioeconomic status, HIV stigma, etc., while religious belief and social support are protective factors for suicide attempts [25–27]. Mesele Wonde’s investigation in Ethiopia found that the risk factors for suicide attempts among adolescents with HIV included opportunistic infection and phase III clinical HIV, so whether to take medication regularly would also affect the suicide attempts of adolescents with HIV [28].

At present, there is no literature report on the incidence and influencing factors of suicide attempts among ethnic minority adolescents with HIV/AIDS in China. Liangshan Prefecture of Sichuan Province has a high incidence of HIV/AIDS, and there are nearly 30,000 HIV/AIDS patients in the whole prefecture [29]. The mental health problems of these patients deserve much social attention . The survey conducted by Yao Yin found that the social support for HIV/AIDS patients in Liangshan Prefecture was poor and needed to be improved [30]. Hui Yang found that HIV patients in Liangshan Prefecture showed a moderate degree of HIV-related stigma [31]. We hope to investigate the prevalence rate and related influencing factors of suicide attempts among Yi adolescents with HIV/AIDS in Liangshan Prefecture, Sichuan Province, China, through a cross-sectional study to improve social and governmental attention to the mental health problems of this group and to carry out targeted intervention measures.

## Objectives and methods

### Objects

From January 2022 to March 2022, the census method was adopted. First, the list of all adolescents with HIV/AIDS in a county of Liangshan Prefecture was collected through the Liangshan Center for Disease Control and Prevention. Then, the anti-HIV center staff in the county hospital sent electronic questionnaires to HIV/AIDS Yi adolescents aged 11–19 years in the county who returned for subsequent visits to investigate their suicide attempts and related factors. A total of 223 Yi adolescent HIV/AIDS patients aged 11–19 were collected in the county, and 180 valid questionnaires were collected, accounting for 80.7% of the total number of this group in the county. A total of 20 eligible adolescent guardians refused our investigation, and another 23 eligible adolescents did not return to the hospital as scheduled.

### Tools

The Adolescent Self-Rating Life Events Check List (ASLEC) was compiled by Xiancheng Liu, with a total of 27 items. They were scored on five scales (1–5 points), where 1 means “the negative life event does not happen, or it does happen but has no impact”; 2 means “the negative life event happens and has a mild impact”; 3 means “the negative life event happens and has a moderate impact”; 4, “the negative life event happens and has a severe impact”; or 5, “the negative life event happens and has an extremely severe impact” [32]. According to the literature description of Xiuhong Xin, the adolescent life events scale was subdivided into 5 factors: punishment factor, loss factor, interpersonal stress factor, academic pressure factor and adaptation factor [33]. The Columbia Suicide Severity Rating Scale (C-SSRS), compiled by Posner in 2011, has a total of six items to assess suicidal ideation and suicide attempts. We selected the sixth suicidal behavior topic to determine whether the subjects had attempted suicide, the question is “Have you ever done anything, started to do anything, or prepared to do anything to end your life? Examples: Collected pills, obtained a gun, gave away valuables, wrote a will or suicide note, took out pills but didn’t swallow any, held a gun but changed your mind or it was grabbed from your hand, went to the roof but didn’t jump; or actually took pills, tried to shoot yourself, cut yourself, tried to hang yourself, etc.” [34, 35].

Self-designed questionnaire survey of teenagers’ gender, age and family economic status (options: less than 1000 RMB/month; 1000–2000 RMB/month; 2000–3000 RMB/month; Higher than 3000 RMB/month), whether they are nuclear families (families with native parents are defined as nuclear families, and families with nonnative parents are defined as nonnuclear families), sleep duration (options of average sleep time per day include: 1) 4 h or less; 2) 5 h; 3) 6 h; 4) 7 h; 5) 8 h; 6) 9 h; 7) 10 h; 8) 11 h; 9) 12 h or more), duration of exercise (average daily exercise of moderate or above intensity options include: 1) less than 1 h; 2) 1 h; 3) 2 h; 4) 3 h; 5) 4 h; 6) 5 h and above), medication for HIV (whether to accept medication regularly), etc.

### Research permission and informed consent

This study was approved by the Ethics Committee of the West China Hospital of Sichuan University. The legal guardians (parents) of the minors were informed of the significance, confidentiality and safety of the study through electronic informed consent and signed the electronic informed consent form. Adhering to the principles of equality, voluntariness and cooperation, only the subjects who agree and sign the informed consent form are investigated. All experiments were performed in accordance with relevant guidelines and regulations (such as the Declaration of Helsinki).

### Statistical processing

All data were analysed using the SPSS 23.0 software package (IBM, Armonk, NY, USA). Descriptive analysis was mainly used to analyse the gender, age, ASLEC score, suicide attempts and other information of the survey samples. Chi-square tests and T-test were used to preliminarily screen the variables related to suicide attempts, and the criteria for screening was p < 0.10, then the potential influencing factors of suicide attempts were further analysed in binary logistic regression. Quantitative data were expressed as the mean ± standard deviation (x ± s), qualitative data were expressed as percentage, two-sided test, the test level = 0.05.

## Results

### Demographic data

There were 223 HIV/AIDS adolescents of Yi nationality aged 11–19 years in the county. In the end, 180 valid questionnaires were collected, accounting for 80.7% of the total number of this group in the county. There were 100 boys (55.6%) and 80 girls (44.4%), with an average age of 13.53 ± 2.032 years (age range 11–19 years). According to the recommendation of the American Sleep Foundation and the Canadian 24-hour Exercise guidelines for children and adolescents, the sleep time of adolescents aged 5–13 should be 9–11 h/night, and the sleep time of adolescents aged 14–17 should be 8–10 h/night [36, 37]. In this study, we defined adolescents aged 13 and younger who slept less than 9 h as insufficient sleep, those who slept more than 9 h as adequate sleep, adolescents aged 14 and older who slept less than 8 h as insufficient sleep, and adolescents aged 14 and older who slept more than 8 h as adequate sleep. According to the recommendations of the WHO, teenagers should spend more than 1 h per day on average exercising at a moderate intensity or above [38], and we define less than 1 h per day exercising at a moderate intensity or above as deficient exercise and reaching 1 h per day as adequate exercise. In this study, 85 adolescents (47.2%) were in the insufficient sleep group, 95 (52.8%) were in the adequate sleep group, 104 (57.8%) were in the deficient exercise group, and 76 (42.2%) were in the adequate exercise group. The statistical results of the ASLEC score and suicide attempts are shown in Table 1, in which the average ASLEC score was 51.54 ± 23.16, and 13.9% (25) of adolescents had suicide attempts.

**Table 1:** Demographic information (number of adolescents/scores, %/X ± S)

Item	Distribution
Gender	
Male	100(55.6%)
Female	80(44.4%)
Age	13.53 ± 2.03 years
Ethnic nationality	
Yi nationality	180(100%)
Family income status	
Less than 1000 RMB/month 1000–2000 RMB/month 2000–3000 RMB/month More than 3000 RMB/month	42(23.3%) 85(47.2%) 39(21.7%) 14(7.8%)
Whether it is a nuclear family (a family with the original parents)	
Yes No	89(49.4%) 91(50.6%)
Whether to take anti-HIV medication regularly	
Yes No	141(78.3%) 39(21.7%)
Sleep	
Insufficient sleep Adequate sleep	85(47.2%) 95(52.8%)
Exercise	
Deficient exercise Adequate exercise	104(57.8%) 76(42.2%)
ASLEC scores*	51.54 ± 23.16
Suicide attempts	
Yes No	25(13.9%) 155(86.1%)

*: Adolescent Self-Rating Life Events Check List

### Research on influencing factors of suicide attempts

The chi-square test and T-test were used to initially screen for variables associated with suicide attempts. We found that factors that may influence suicide attempts included age and ASLEC scores, while the remaining factors, including sex, nuclear family status, regular medication acceptance, sleep, exercise, and family economic status, were not significantly correlated with suicide attempts. Binary logistic regression was used to further evaluate the effects of age and ASLEC scores on suicide attempts, and the obtained logistic model was statistically significant (x^2^ = 26.352, p < 0. 001). The model was able to correctly classify 85.6% of the subjects, with a sensitivity of 12.0% and specificity of 97.4%. ASLEC scores were found to be a risk factor for suicide attempts (OR = 1.047, p < 0.001, 95% CI 1.027–1.067) (see Model 1 in Table 2). Of the ASLEC factors, adaptation was a risk factor for suicide attempts (OR = 1.203, p = 0.026, 95% CI 1.022–1.416). The academic pressure factor showed a tendency to be a risk factor for suicide attempts (OR = 1.149, p = 0.077, 95% CI 0.985–1.339), while the punishment factor, interpersonal stress factor and loss factor had no significant statistical correlation with suicide attempts (see Model 2 in Table 2).

**Table 2:** Binary logistic regression analysis of suicide attempt-related factors

Variables	B	SE	Wald	OR(95%CI)	p
Model 1					
Constant Age ASLEC scores	-3.061 -0.071 0.046	1.625 0.120 0.010	4.913 0.351 22.174	0.027 0.931(0.736, 1.179) 1.047(1.027, 1.067)	= 0.027 = 0.553 < 0.001
Model 2*					
Constant	-4.686	1.755	7.130	0.009	= 0.008
Age	0.001	0.127	0.000	1.001(0.780, 1.284)	= 0.995
Interpersonal stress factor	0.029	0.102	0.078	1.029(0.842, 1.257)	= 0.780
Academic pressure factor	0.139	0.078	3.130	1.149(0.985, 1.339)	= 0.077
Punishment factor	-0.007	0.055	0.016	0.993(0.892, 1.106)	= 0.900
Loss factor	-0.036	0.063	0.325	0.965(0.853, 1.092)	= 0.569
Adaptation factor	0.185	0.083	4.936	1.203(1.022, 1.416)	= 0.026

* ASLEC (Adolescent Self-Rating Life Events Check List) has five factors: interpersonal stress factor, academic pressure factor, punishment factor, loss factor and adaptation factor

## Discussion

Our study reports for the first time the incidence of attempted suicide among Yi adolescents with HIV/AIDS in Liangshan Prefecture, Sichuan Province, at 13.9%, lower than the findings of Anthony An Olashore in Botswana [39] and close to the findings of Jayraan Badiee in America [40]. On the whole, the problem of suicide attempts among Yi adolescents with HIV/AIDS in Liangshan Prefecture is prominent and needs to be given sufficient attention.

According to the study of Lily A. Brown, the risk factors for suicide in HIV patients include major depression, posttraumatic stress disorder and substance dependence [41]. A 16-year longitudinal study conducted by Philip Kreniske in New York found that anxiety, mood disorders, negative life events, and HIV-related stigma were associated with an increased risk of suicide attempts, while high individual and family self-concept had a protective effect [42]. S. Ashaba’s study of HIV adolescents in Uganda showed that adolescent suicide was significantly associated with maltreatment and HIV stigma [43]. We found that the influencing factors of suicide attempts among HIV/AIDS adolescents of Yi nationality in Liangshan Prefecture were negative life events, among which adaptation was a risk factor for suicide attempts, and academic pressure showed a tendency to become a risk factor for suicide attempts. Adolescents with poor adaptation in life often adopt unhealthy coping strategies to deal with stressful life events. Sun Mi Kim found that unhealthy coping strategies were significantly correlated with suicide attempts among adolescent girls in South Korea, and Fang Tang also found similar results in Chinese adolescents [44, 45]. Adaptive factors include changes in lifestyle habits, hate of going to school, bad or broken love relationships, long distance from family members for a long time, and tension with teachers. The risk of suicidality of American children from the Adolescent Brain and Cognitive Development (ABCD) study reduced with greater parental supervision and positive school involvement [46]. We believe that family members should not stay away from these young people for a long time. Parents should pay more attention to the relationship between adolescents and teachers, whether they are willing to go to school and whether there is a bad love relationship, etc. If there is some problem, they should deal with it actively, for example, communicate with their teacher when their relationship is tense to prevent the occurrence of suicidal behaviors caused by adolescent’s negative coping styles. P. Mortier’s investigation in Belgium and Lan Guo’s study in China both found a significant correlation between adolescents’ academic performance and suicide attempts [47, 48]. In this study, no significant correlation was found between age, gender, family economic status, sleep status, exercise status, nuclear family status, regular medication acceptance and suicide attempts. According to our survey results, we need to pay special attention to adolescents with HIV/AIDS in Liangshan Prefecture who have poor adaptability and high academic pressure.

Our study also has some limitations. First, our study was a cross-sectional study using the self-rating scale, which could not infer causality, and the self-rating scale may produce a bias due to the difference in the literacy and intelligence level of the subjects. In the future, longitudinal cohort studies, the Pittsburgh Sleep Quality Index and wearable motion measuring instruments are needed to conduct more objective measurements of sleep and exercise, and larger sample studies are needed to further explore the above issues.

In conclusion, we report the incidence of suicide attempts and its related influencing factors in HIV/AIDS adolescents of Yi nationality in Liangshan Prefecture, Sichuan Province, China. We find that the incidence of suicide attempts is high in this group, and negative life events are a risk factor for suicide attempts. The mental health status of this group needs to be considered by all walks of life in our society, especially adolescents with negative life events. We hope to expand the sample size in future studies and conduct longitudinal follow-up studies for further exploration and hope that our work can help this group obtain more targeted interventions to improve their clinical suffering and prognosis.

## Data Availability

The datasets generated and/or analysed during the current study are not publicly available due [Data is not shared to protect the subjects’ privacy.] but are available from the corresponding author on reasonable request.
